# Linoleate appears to protect against palmitate-induced inflammation in Huh7 cells

**DOI:** 10.1186/1476-511X-13-78

**Published:** 2014-05-13

**Authors:** Hitoshi Maruyama, Masanori Takahashi, Tadashi Sekimoto, Taro Shimada, Osamu Yokosuka

**Affiliations:** 1 Department of Gastroenterology, Chiba University Graduate School of Medicine, 1-8-1, Inohana, Chuou-ku 260-8670, Chiba, Japan

**Keywords:** Linoleate, Palmitate, Hepatocyte, Interleukin-8, Proinflammatory cytokine, Nonalcoholic steatohepatitis

## Abstract

**Background:**

Polyunsaturated fatty acids (PUFAs) may protect against metabolic diseases. Although the benefits of the n-3 family of PUFA have been well investigated in nonalcoholic steatohepatitis (NASH), little is known about the effect of the n-6 family. This study examined the effect of linoleate, a member of the n-6 family, on regulation of the palmitate-induced inflammatory cytokine interleukin-8 (IL8) in hepatocytes.

**Methods:**

Huh7 cells and HepG2 cells were cultured with and without free fatty acid treatment (palmitate and linoleate, alone or in combination, 100–1000 μM). Inflammatory pathways, lipid accumulation, apoptosis and cell viability were monitored.

**Results:**

Dose- and time-related changes of IL8 mRNA expression were examined and 9 h treatment with 500 μM palmitate showed the greatest elevation of IL8. Co-treatment with 500 μM palmitate and 400 μM linoleate significantly suppressed IL8 production below that with palmitate alone in both cells (both mRNA and protein). A quantitative measurement for lipid accumulation showed no significant difference between palmitate-treated cells (1.69 ± 0.21), linoleate-treated cells (1.61 ± 0.16) and palmitate and linoleate-treated cells (1.73 ± 0.22, NS, n = 7). The co-treatment with 400 μM linoleate inhibited phospho-c-Jun N-terminal kinase (pJNK) activation and IkBα reduction caused by 500 μM palmitate treatment. Treatment with 400 μM linoleate alone led to IL8 production (5.48 fold change), similar to co-treatment, with no influence on the expression of pJNK/IkBα. The cell viability was similar between treatment with 500 μM palmitate and with both 500 μM palmitate and 400 μM linoleate, showing no significant changes in the expression of cleaved caspase-3.

**Conclusions:**

Linoleate is a potent regulator of the proinflammatory cytokine IL8 via the JNK and nuclear factor kappa B pathways that are involved in the pathophysiology of NASH, suggesting a future recommendation of dietary management.

## Background

Nonalcoholic fatty liver disease (NAFLD) is increasing worldwide as a leading cause of chronic liver damage [1,2]. It is closely associated with obesity, diabetes and hyperlipidemia [3-5]. Insulin resistance, endoplasmic reticulum (ER) stress, inflammation, and oxidative stress have been identified as underlying features in the development of nonalcoholic steatohepatitis (NASH), although the precise mechanisms remain unclear [6,7].

Elevated free fatty acids (FFAs) are considered to be a major cause of injury and death of liver cells in NASH [7,8]. Recent studies have shown differences between controls and patients with NAFLD/NASH in the content of FFA in the serum and liver [9,10], probably caused by an imbalance of dietary intake and/or impaired metabolism [11]. Furthermore, changes in the FFA content of the liver may affect lipid metabolism and inflammation.

Influences of saturated fatty acids, as typified by palmitate, may account for the liver cell damage which contributes to developing NASH, characterized by saturated FFA-induced lipotoxicity [8], lipoapoptosis [12] and insulin resistance [13]. Inflammation may also be a major cause of NASH. Lipopolysaccharide (LPS), a gut-derived toll-like receptor 4 ligand, enhances liver injury and increases inflammatory cytokine induction in a NASH model, suggesting the features of a first and second hit leading to the development of NASH [14]. Another study reported that palmitate activates the inflammasome and induces sensitization to LPS-induced interleukin 1β (IL1β) release by hepatocytes [15]. Furthermore, palmitate triggers the release of danger signals from hepatocytes in a caspase-dependent manner.

A production of IL8, a proinflammatory cytokine, may be closely involved in the pathogenesis of NASH [16]. IL-8 is produced in response to inflammatory mediators, such as IL1α, IL1β, and tumor necrosis factor (TNF) in hepatocytes [17], and its production is dependent on nuclear factor kappa B (NFkB) and c-Jun N-terminal kinase (JNK) [16]. Palmitate-induced IL8 production may also be closely associated with the development of diabetes [18]. Palmiate also induces IL-6 via NFkB in adipocytes [19].

The n-3 and n-6 polyunsaturated fatty acids (PUFAs) offer protective effects against metabolic abnormalities [9]. The benefits of n-3 family of PUFA are well-known, they protect against the adverse symptoms of metabolic syndrome and reduce the risk of heart disease [20]. Meanwhile, recent double-blind study has shown that daily supplementation with 1 g of n-3 FAs did not reduce the rate of cardiovascular events in patients at high risk for the event during a median follow-up periods of 6.2 years [21]. Anyhow, there are many studies which focused on the effects of n-3 PUFA. However, little is known about the effects of the n-6 family of PUFA against metabolic diseases. Linoleate is a member of the n-6 family and a major component of plant oil; sunflower, soybean and safflower oils contain more than 50% linoleate [22]. It is also present in fish oil, along with the omega-3 family which is the principal component [23].

Here, we have designed a study to explore the potential effect of linoleate in the regulation of the inflammatory cytokine IL8 in Huh7 cells and HepG2 cells. The aims of the study were: 1) to examine the dose-dependent effect of linoleate in the regulation of this proinflammatory cytokine, 2) to examine the effect of linoleate on the palmitate-induced lipid accumulation, 3) to determine the cellular outcomes of co-treatment with palmitate and linoleate 4) and to determine the pathways involved in the regulation of the palmitate-induced proinflammatory cytokine by linoleate treatment.

## Results

### IL8 expression in FFA treated Huh-7 cells

#### Time and dose dependent changes of IL8 expression after palmitate treatment

**Figure 1:**
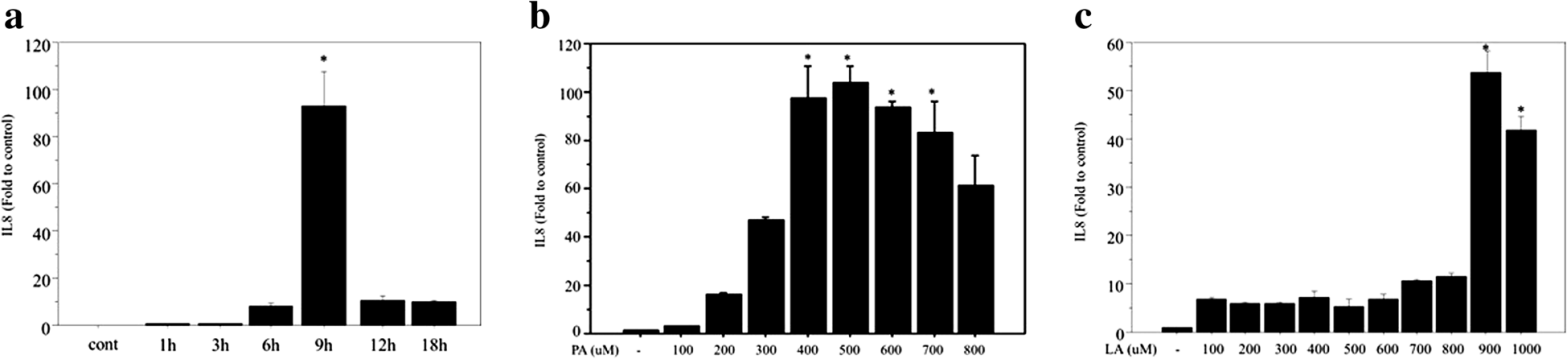
**Dose- and time-related changes of IL8 mRNA expression following free fatty acid treatment. 1a**. Expression of IL8 following 500 μM palmitate treatment, showing the highest expression with 9 hours of treatment (92.1 fold greater than the control, Huh7 cells incubated with bovine serum albumin; n = 4, *p < 0.0001). **b**. Expression of IL8 after 9 hours of treatment with palmitate, showing dose-related changes with the highest expression of IL8 following treatment with 500 μM palmitate (104.9 fold greater than the control, Huh7 cells incubated with bovine serum albumin; n = 4, *p < 0.01). **c**. Expression of IL8 after 9 hours of treatment with linoleate, showing dose-related changes. Expression of IL8 was 5.2 to 11.7 fold increase from 100 to 800 μM, however, it increased markedly at 900 μM with a 53.8 fold increase and 1000 μM with a 41.9 fold increase (n = 4, *p < 0.01).

Time- and dose-related changes of IL8 in HepG2 cells were reported in a previous study [16]. The current study detected the time-related changes of IL8 mRNA expression in Huh7 cells following treatment with 500 μM palmitate, this dose being based on data in the literatures [24,25]. IL8 expression was highest after 9 hours of treatment, being 92.1 fold greater than the control (Huh7 incubated with bovine serum albumin) (Figure 1A). IL8 expression after 9 hours of palmitate treatment showed dose-related changes, the highest expression being with 500 μM palmitate treatment (104.9 fold greater than the control) (Figure 1B).

#### Dose dependent changes of IL8 after linoleate treatment

According to the results of palmitate treatment, IL8 mRNA expression due to linoleate treatment was also assessed after 9 hours of treatment. IL8 expression increased 5.2 to 11.7 fold with doses from 100 to 800 μM, however, it increased markedly by 53.8 fold at 900 μM and 41.9 fold at 1000 μM (Figure 1C). No expression of TNFα mRNA was detected in cells treated with palmitate or linoleate (Data are not shown).

### Lipid accumulation in Huh7 cells after 9 hours of treatment with FFA

**Figure 2:**
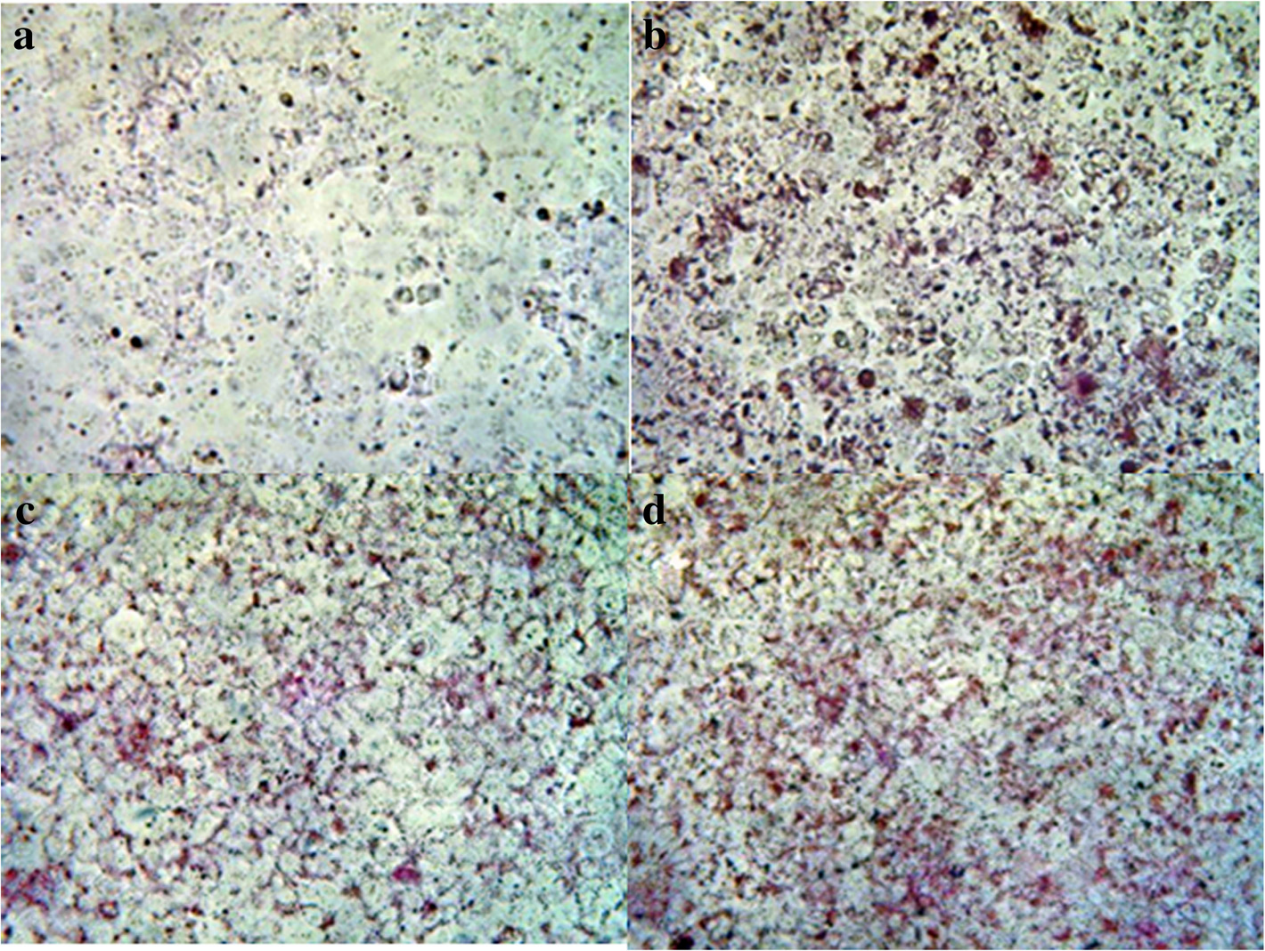
**Lipid accumulation in Huh7 cells.** Lipid accumulation was detected in Huh7 cells by staining with Oil Red O. The images were captured by microscopy at a magnification of 20×. **a**. Untreated cells. **b**. Cells after 9 hours of treatment with 500 μM palmitate. **c**. Cells after 9 hours of treatment with 400 μM linoleate. **d**. Cells after 9 hours of treatment with both 500 μM palmitate and 400 μM linoleate.

Lipid accumulation was detected in Huh7 cells by staining with Oil Red O. The images were captured by microscopy at a magnification of 20× (Figure 2).

A quantitative measurement for lipid accumulation showed no significant difference between palmitate-treated cells (1.69 ± 0.21), linoleate-treated cells (1.61 ± 0.16) and palmitate and linoleate-treated cells (1.73 ± 0.22, NS, n = 7).

### Changes of IL8 expression after co-incubation with palmitate and linoleate in Huh-7 cells and HepG2 cells

**Figure 3:**
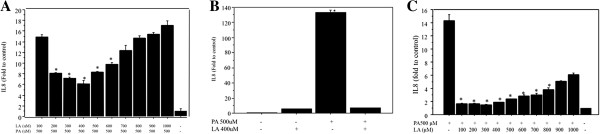
**Changes of IL8 mRNA expression following co-incubation with palmitate and linoleate. A**. Dose-related changes of IL8 expression following co-incubation with 500 μM palmitate and linoleate (100 to 1000 μM) in Huh7 cells. Dose-related effect of linoleate in the regulation of palmitate-induced IL8 production was assessed following 9 hours of incubation. A 400 μM linoleate showed maximum suppression (n = 4, *p < 0.05). **B**. Suppressive effect of linoleate against IL8 production induced by palmitate in Huh7 cells. IL8 mRNA expression was significantly lower following co-incubation with palmitate and linoleate (7.33 fold increase; greater than the control, Huh7 cells incubated with bovine serum albumin) than those with palmitate treatment alone (133.5 fold increase, n = 6, *p < 0.0001). A co-incubation with 400μM linoleate showed suppression of IL8 expression with 94.5% decrease compared to the cells treated by 500μM palmitate alone. **C**. Dose-related changes of IL8 expression following co-incubation with 500 μM palmitate and linoleate (100 to 1000 μM) in HepG2 cells. Dose-related effect of linoleate in the regulation of palmitate-induced IL8 production was also assessed following 9 hours of incubation in HepG2 cells. A 300 μM linoleate showed maximum suppression with 87.1% decrease compared to the IL8 expression in the cells treated by 500 μM palmiate alone (n = 4, *p < 0.01).

The dose-related effect of linoleate in regulating palmitate-induced IL8 production was assessed by 9 hours of co-incubation with 500 μM palmitate and 100–1000 μM linoleate (Figure 3A). The expression of IL8 mRNA showed dose-related effects from 5.6 to 17.9 fold above the control (Huh7 incubated with bovine serum albumin) and 400 μM linoleate provided the most significant effect in suppressing IL8 expression (5.6 fold change). Increased IL8 expression was significantly suppressed by co-treatment with palmitate and linoleate (7.33 fold change) compared to treatment with palmitate alone (133.5 fold change, p < 0.0001, n = 6) (Figure 3B) in Huh7 cells. Treatment with 400 μM linoleate alone increased IL8 expression (5.48 fold change) to a level similar to that with co-treatment in Huh7 cells.

As the increase of IL8 according to the incubation time and dose of palmitate treatment in HepG2 cells is well-known [16], the effect to co-incubation with palmitate and linoleate on the IL8 expression was also examined in HepG2 cells. The suppression of IL8 mRNA was detected by the addition of linoleate (Figure 3C). A 300 μM linoleate showed maximum suppression with 87.1% decrease compared to the IL8 expression in the cells treated by 500 μM palmiate alone.

### Production of IL8 due to the FFA treatment

**Figure 4:**
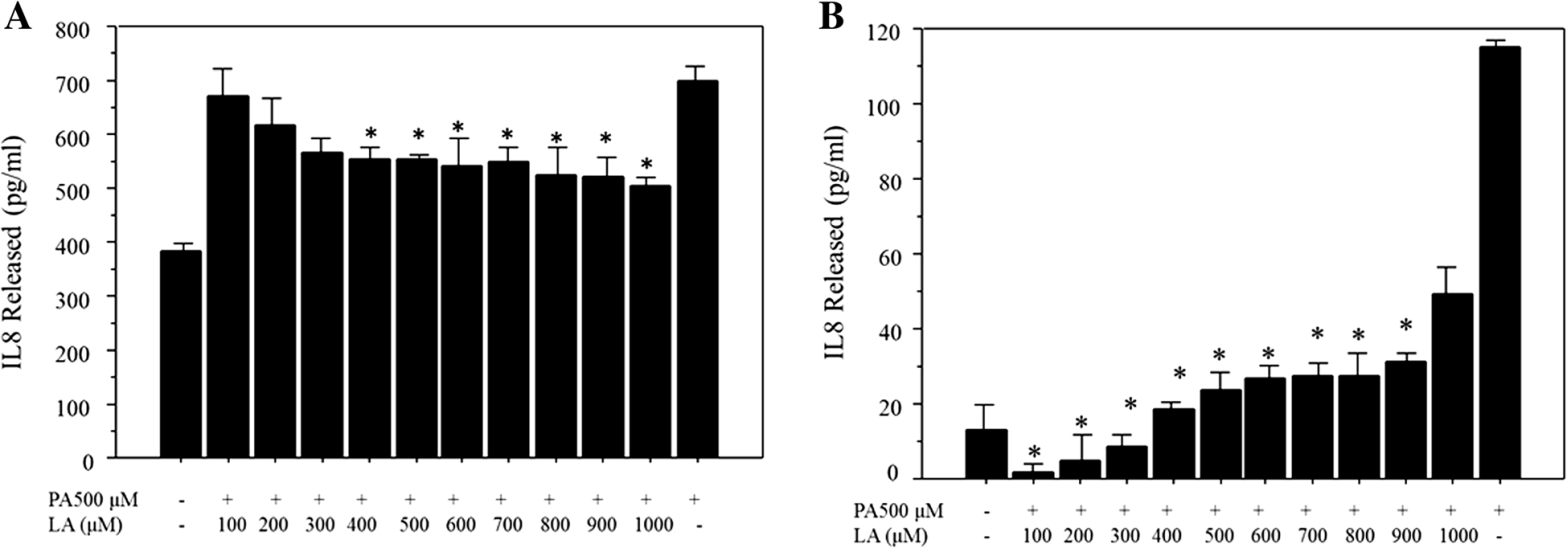
**Assessment of IL8 production due to free fatty acid treatment by commercial ELISA kit. A**. IL8 production in Huh-7 cells. The IL8 production was significantly reduced by the co-incubation with linoleate compared to the cells treated by palmitate alone; with 400-1000 μM linoleate in the Huh7 cells. The data represent a mean value of 4 experiments ± standard deviation. *p = 0.041 compared with cells treated by palmitate alone. **B**. IL8 production in HepG2 cells. The IL8 production was significantly reduced by the co-incubation with linoleate compared to the cells treated by palmitate alone; with 100-900 μM linoleate in the HepG2 cells. The data represent a mean value of 4 experiments ± standard deviation.*p = 0.011 compared with cells treated by palmitate alone.

The IL8 production was assessed by enzyme-linked immunosorbent assay (ELISA) in both Huh-7 cells and HepG2 cells. The IL8 release increased by palmitate treatment alone compared to untreated cells. The IL8 production was significantly reduced by the co-incubation with linoleate compared to the cells treated by palmitate alone; with 400-1000 μM linoleate in the Huh7 cells, and with 100-900 μM linoleate in the HepG2 cells (Figure 4A, B).

### Cell viability with respect to FFA treatment in Huh7 cells

There was no significant difference in the viability between cells treated with 500 μM palmitate alone (75 ± 5.6%) and cells treated by co-incubation with 500 μM palmitate and 400 μM linoleate (69.8 ± 7.7%; n = 6, p = 0.21). However, the viability of cells treated with a high concentration of linoleate (28.7 ± 9.9% at 900 μM; 29.2 ± 4% at 1000 μM) was significantly lower than that of cells treated with a low concentration of linoleate (71.4 ± 4.8 at 100 μM; 63.3 ± 15.9% at 200 μM, n = 6, p < 0.0001).

### Apoptosis following FFA treatment

**Figure 5:**
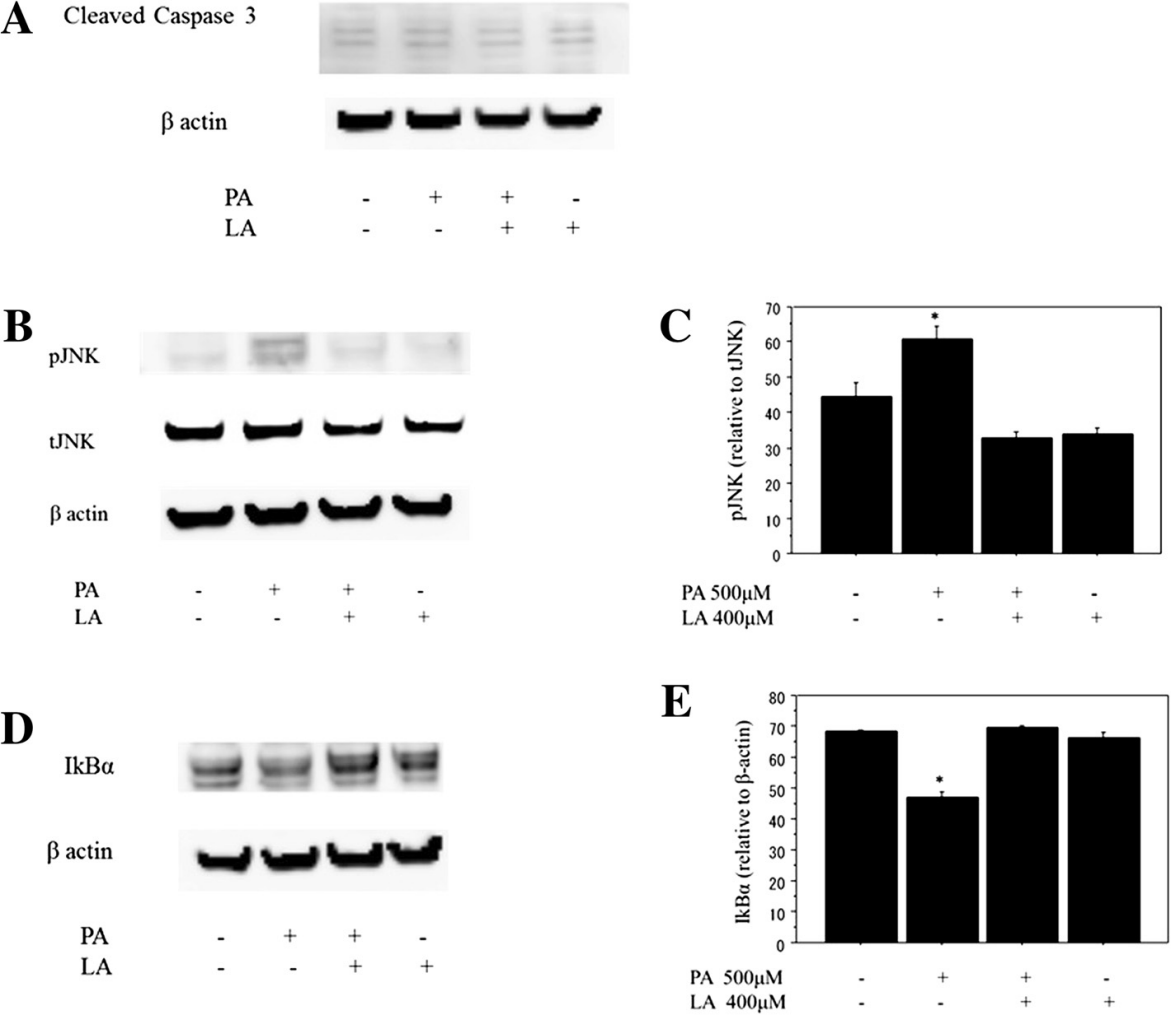
**Analysis of protein expression by western blotting. A**. Cleaved caspase-3. Caspase-3 was used as an indicator of apoptosis in Huh-7 cells and cleaved caspase-3 was examined after free fatty acid treatment to determine the presence of apoptosis at a molecular level. The cleaved caspase-3 (17 kDa and 19 kDa) showed no significant changes in the expression after the FFA treatment (n = 4), among cells treated by 500 μM palmitate, 400 μM linoleate and co-incubation with both of them. **B**, **C**, **D**, **E**. The effect of co-incubation with palmitate and linoleate on markers of inflammation. Huh7 cells were incubated with bovine serum albumin (control) or after free fatty acid treatment (500 μM palmitate and/or 400 μM linoleate). Western blot analysis of phospho-c-Jun N-terminal kinase and total-c-Jun N-terminal kinase or β-actin (Figure 5B, 6 hr incubation), IkBα (Figure 5D, 3 hr incubation) and β-actin (internal control). The gels shown are representative of 4 independent experiments and data in the graphs are expressed as the ratio of the target protein to total-c-Jun N-terminal kinase or β-actin (Figure 5C and 5E). The expression of pJNK increased significantly in Huh-7 cells treated with 500 μM palmitate (64.9 ± 4.1) compared to control cells (35 ± 4.2; *p<0.05), cells treated with both 500 μM palmitate and 400 μM linoleate (32.3 ± 2.5; *p<0.01), and cells treated with 400 μM linoleate (36.1 ± 3.3; *p<0.01; n = 4). The expression of IkBα decreased significantly in Huh-7 cells treated with 500 μM palmitate (46.6 ± 2.2) compared to control cells (68.1 ± 5.9 ; *p<0.05), cells treated with both 500 μM palmitate and 400 μM linoleate (68.3 ± 5.7; *p<0.05), and cells treated with 400 μM linoleate (65.7 ± 3.3; *p<0.05; n = 4).

Caspase-3 was used as an indicator of apoptosis of Huh-7 cells and cleaved caspase-3 was examined to determine at a molecular level the presence of apoptosis after FFA treatment. Cleaved caspase-3 showed no significant changes in the expression after the FFA treatment (n = 4), among cells treated by 500 μM palmitate, 400 μM linoleate and co-incubation with both of them (Figure 5A).

### Changes in inflammatory pathways following FFA treatment

Previous studies have reported that pJNK activation and reduced IkBα expression are key mechanisms for palmitate-induced IL8 production [16,26]. Therefore, the present study examined the relevant pathways in Huh7 cells and found the similar findings (Figure 5B, C, D, E). Here, analysis of cytoplasmic extracts showed that co-incubation with 500 μM palmitate and 400 μM linoleate significantly inhibited pJNK activation and the reduction of IkBa expression, indicating the anti-inflammatory effect of linoleate. However, treatment with 400 μM linoleate alone did not affect the expression of pJNK/IkBa.

## Discussion

Detailed and comprehensive analysis of the effects of FFA on cellular pathways may provide a significant insight into the study of NASH, a disorder caused by complicated mechanisms. Among the complex pathways leading to NASH, inflammation features as a major factor in the pathogenesis of steatohepatitis [27].

The current study proved the value of linoleate as a potent protector against lipotoxicity-related inflammation. First, we reported the protective and dose-dependent effect of co-incubation with linoleate against the increased production of IL8 that follows palmitate treatment, showing no additional effect on lipid accumulation. Second, we determined the lipotoxicity of an overdose of linoleate (more than twice that dose giving the most beneficial effect), showing IL8 production from Huh7 cells. Third, the study showed that linoleate has anti-inflammatory effects, with the regulation of JNK/NFkB pathways.

A recent study has shown the effect of unsaturated fatty acids against NLRP3 inflammasome [28], suggesting the potential function of unsaturated fatty acids to prevent inflammation.

It is clear that PUFAs provide a beneficial effect by suppressing lipogenesis and enhancing FFA oxidation [20], as dietary n-6 and n-3 PUFA reduce triglyceride accumulation in skeletal muscle and, potentially, in cardiomyocytes and beta cells [29]. PUFAs also reduce the production of hepatic malonyl coenzyme A (CoA), which is a negative metabolic effector of carnitine palmitoyltransferase [30]. In particular, the n-3 PUFAs downregulate the transcription factor sterol regulatory element binding protein-1 (SREBP-1), which promotes triglyceride accumulation in the liver by the up-regulation of lipogenic genes such as fatty acid synthase (FAS) and sterol Co-A desaturase-1 (SCD-1). In fact, diets rich in 18 : 2 (n-6) or 20 : 5 and 22 : 6 (n-3) were found to lead to a reduction of the hepatic nuclear and precursor content of mature SREBP-1 by 85% and 60%, respectively [31]. It is also known that PUFAs upregulate peroxisomal proliferator activated receptor-α (PPAR-α), which stimulates hepatic fatty acid oxidation and activates fatty acid transport protein and acyl-CoA synthase. This effect is more potent with the n-3 family than the n-6 family. Altogether, these studies suggest that the effects of the n-3 family seem to be dominant over those of the n-6 family in coordinating the upregulation of lipid oxidation and downregulation of lipid synthesis. Actually, macrosteatosis constitutes a feature of NAFLD, which is characterized by the accumulation of triglycerides in the liver together with a reduced hepatic content of n-3 long chain PUFAs and an abnormally high n-6 : n-3 PUFA ratio [32].

Contrary to the beneficial aspect of PUFAs, a negative effect of linoleate has been reported on H4IIE cells [33]. The study showed that linoleate promotes apoptosis through the release of cytochrome C but not cleavage of caspase-3. The authors suggested that ER stress may contribute to fatty acid-induced apoptosis of liver cells. However, our study showed no significant difference in the viability between cells with and without linoleate treatment, showing no activation of caspase-3. This may be due to the difference of cells and doses of FFA, their study used rat liver cells treated with 125–250 μM palmitate and linoleate whose concentration was lower than that in our study.

Investigators have studied the effect of incubation with a combination of saturated FFA and PUFA. Wei et al. reported that co-incubation with oleate reduced or prevented the induction of ER stress and cell death caused by palmitate [24]. The authors suggested that the phenomenon may be attributable to alternations in the trafficking of saturated fatty acids away from the ER membrane in the presence of unsaturated fatty acids. Another study showed that co-incubation of palmitate and 125 μM linoleate significantly decreased apoptosis, compared to palmitate treatment alone [33]. Meanwhile, the authors of the latter study also mentioned that the effect was not observed with a dose of 250 μM linoleate, indicating the dose-dependent effect of unsaturated FFA. Anyhow, these data may offer a positive synergistic effect of unsaturated fatty acids under conditions of co-incubation, similar to the results in our study.

It is recommended to take saturated fat <10% of the total caloric intake for the improvement of plasma lipid levels [34]. As for the PUFA, supplementation with pharmacological doses of n-3 fatty acids (>2–3 g/day) reduces triglyceride levels, but a higher dosage may increase low-density lipoprotein cholesterol [34,35]. Effect of n-3 fatty acids on the event of ischemic heart disease may be controversial [21,36]. Meanwhile, a recent evidence has shown that the consumption of at least 5% to 10% of energy from omega-6 PUFAs reduces the risk of coronary heart disease relative to lower intakes, and suggested that higher intakes appear to be safe and may be even more beneficial [37]. Although it has been considered that care should be taken to achieve a high n-6 : n-3 ratio in the diet and a ratio "1-4 : 1" of n-6 to n-3 in the diet may be within the healthy range [38], not enough data are available to make a recommendation regarding the optimal n-3/n-6 fatty acid ratio at present [34].

In summary, the current study has shown the potent anti-inflammatory effect of linoleate via JNK/NFkB pathways which are closely involved in the pathophysiology of NASH. Unfortunately, however, the study condition may not be physiological with relatively high concentration of FFA. Further study may be needed to unveil the role of FFA comprehensively, resulting in a future recommendation for dietary management.

## Methods

### Cell culture

Huh7 cells and HepG2, a human hepatoma cell line, were used for the study. They were cultured in Dulbecco’s Modified Eagle’s Medium (DMEM) supplemented with 10% heat-activated fetal bovine serum, 100 IU/ml penicillin and 100 μg/ml streptomycin.

### FFA treatment

FFA, palmitate and linoleate, were purchased from Sigma Chemical Company (St. Louis, MO). Each fatty acid was complexed with 5% bovine serum albumin and incubated with the cells at final concentrations of 100–1000 μM. FFA treatment was performed by palmitate (1 to 18 h, 100–800 μM) or linoleate alone (9 h, 100–1000 μM), or co-incubation with both of them (9 h, palmitate 500 μM and linoleate 100-1000 μM).

### Staining with Oil Red O

Accumulation of fatty acids was detected by staining with Oil Red O (Wako, 154–02072). Huh7 cells were incubated in the 6-well plates overnight and treated with FFA. Dishes with control cells (cells without FFA treatment) and FFA-treated cells were washed with phosphate buffered saline (PBS) and fixed in 10% formalin. After washing by PBS, Oil Red O was added.

### Cell viability

The cell viability was determined using the CellTiter 96 AQueous One Solution Cell Proliferation Assay (MTS, Promea, G3580). After overnight incubation in 96 well plates with 20,000 cells/well, the cells were washed twice with PBS and treated with FFA for 9 hours. The MTS reagent was added to the wells at a 6:1 ratio of medium to MTS solution and optical densities were measured at 490 nm after 2 hr incubation. The viability was expressed as a ratio (%) of FFA-treated cells to control (untreated cells).

### Human IL-8 Assay

IL-8 was measured in cell-free culture supernatants using highly specific ELISA kit purchased from Diaclone SAS (Besancon Cedex, France), according to the manufacturer’s protocol.

### Quantification of lipid accumulation

Accumulated lipid was quantitatively assessed using Steatosis Colorimertic Assay kit (Cayman Chemical Company, MI). After overnight incubation of 10,000 cells/well in 96 well plates, the cells were washed twice with PBS and treated with FFA. The 9 h-treated cells were stained according to the manufacture’s protocol, and lipid accumulation was determined by the absorbance at 490 nm. The lipid accumulation was expressed as a ratio of FFA-treated cells to control (untreated cells).

### Real-time quantitative reverse transcription polymerase chain reaction

Total cellular RNA was extracted using TRIzol reagent according to the manufacturer’s protocol (Invitrogen, Carlsbad, CA). Single-strand cDNAs were synthesized from 2 μg total RNA in a 20 μL reaction (SuperScript® VILO™, cDNA Synthesis Kit, Invitrogen). Polymerase chain reactions (PCR) were performed using cDNA, SYBR green (Platinum® SYBR® Green qPCR SuperMix-UDG with ROX, Invitrogen) and primers for IL8, TNFα, and glyceraldehyde-3-phosphate dehydrogenase (GAPDH), purchased from Takara Bio (Tokyo, Japan; Table 1). Reactions were run in triplicate and data were calculated as the change in cycle threshold (ΔCT) for the target gene relative to the ΔCT for GAPDH (endogenous control).

**Table 1:** Primers for quantitative polymerase chain reaction

**Gene**	**Forward/reverse**	**Sequence 5′-3′**
IL8	Forward	ACACTGCGCCAACACAGAAATTA
	Reverse	TTTGCTTGAAGTTTCACTGGCATC
TNFα	Forward	GTGACAAGCCTGTAGCCCATGTT
	Reverse	TTATCTCTCAGCTCCACGCCATT
GAPDH	Forward	GCACCGTCAAGGCTGAGAAC
	Reverse	TGGTGAAGACGCCAGTGGA

### Protein extraction and western blot analysis

Cell lysates were centrifuged at 12,000 g for 15 min and proteins in the supernatants were used for western blotting to detect cleaved caspase-3. Cytoplasmic extraction was conducted using NE-PER®Nuclear and Cytoplasmic Extraction Reagents (Thermo scientific, Pierce Biotechnology, Inc., Rockford, IL) with protease inhibitor and phosphatase inhibitor (Sigma Chemical Company, St. Louis, MO). Cytoplasmic extracts of the cells were used for western blotting to detect phospho-c-Jun N-terminal kinase (pJNK), total JNK (tJNK) and IkBα.

Proteins were separated using 4% -12% NuPAGE® Novex Bis-Tris Mini Gels (Invitrogen) and were transferred to a nitrocellulose membrane for 1.5 hr at 40 V using a western blot apparatus (Invitrogen). After overnight incubation with primary antibody, the membranes were washed and were incubated with horseradish peroxidase-conjugated secondary antibodies (Pierce Biotechnology, Inc., Rockford, IL).

Proteins were detected with an enhancement using SuperSignal chemiluminescence reagent (Pierce) and a LAS-4000UV (Fuji Film, Tokyo, Japan). Primary antibody (Cleaved Caspase-3, IkBα, pJNK, tJNK), secondary antibody and β-actin were purchased from Cell Signaling (Beverly, MA).

### Statistical analysis

Continuous variables were compared by the Student’s t-test or Fisher’s Protected Least Significant Difference test. *P*-values less than 0.05 were considered statistically significant in all analyses. Statistical analysis was performed using the Dr. SPSS software package (version 11.0 J for Windows; SPSS Inc., Chicago, Illinois, USA).

### Ethical approval

The study protocol was approved by the Ethical Committee of Chiba University.

## Abbreviations

NAFLD: Nonalcoholic fatty liver disease; ER: endoplasmic reticulum; NASH: nonalcoholic steatohepatitis; FFA: free fatty acids; IL8: interleukin-8; TNF: tumor necrosis factor; NFkB: nuclear factor kappa B; JNK: c-Jun N-terminal kinase; PUFA: polyunsaturated fatty acid; DMEM: dulbecco’s Modified Eagle’s Medium; PBS: phosphate buffered saline; ELISA: enzyme-linked immunosorbent assay; PCR: polymerase chain reaction; GAPDH: glyceraldehyde-3-phosphate dehydrogenase; ΔCT: cycle threshold; pJNK: phospho-c-Jun N-terminal kinase; LPS: lipopolysaccharide; CoA: coenzyme A; SREBP-1: steroal regulatory element binding protein-1; FAS: fatty acid synthase; SCD-1: sterol Co-A desaturase-1; PPAR-α: peroxisomal proliferator activated receptor-α.

## Competing interests

The authors declare that they have no competing interests.

## Authors’ contributions

HM performed the research. HM, MT, TSek, and TShi collected and analyzed the data. HM designed the research study and wrote the paper. OY contributed to the design of the study. All authors read and approved the final manuscript.
